# Artificial intelligence as the More Knowledgeable Other (MKO) 2.0: metadata-driven adaptive scaffolding for narrative comprehension

**DOI:** 10.3389/frai.2026.1933178

**Published:** 2026-10-28

**Authors:** Patrick O. Akinwumi, Meihua Qian, Itunu Olaniran Akande, Oyinkansola A. Babatope

**Affiliations:** 1 College of Education, Clemson University, Clemson, SC, United States; 2 Business School, Bournemouth University, Poole, United Kingdom

**Keywords:** artificial intelligence in education, educational NLP, hint generation, instructional scaffolding, large language models, narrative understanding, reading comprehension

## Abstract

**Introduction:**

Recent advances in large language models (LLMs) have expanded the capabilities of artificial intelligence (AI) in education, yet much of this progress remains focused on answer generation rather than theoretically grounded instructional support. This study introduces *AI as the More Knowledgeable Other (MKO) 2.0*, an MKO-inspired framework for generating task-sensitive scaffolds from structured question metadata.

**Methods:**

Using the Quest Dataset of 6,989 narrative-comprehension questions, a rule-based system operationalized three task dimensions, comprehension attribute, explicit/implicit evidence status, and local/summary scope, into seven primary scaffold families and 28 surface realizations. An attribute-stratified sample of 140 questions was subsequently used to compare rule-based scaffolds with generic narrative-grounded and MKO-conditioned LLM scaffolds.

**Findings:**

Across paired LLM outputs, MKO-conditioned scaffolds were more elaborated than generic scaffolds, averaging 5.18 additional words per instance (*W* = 356.5, *p* < 0.001), and showed slightly higher corpus-level lexical variation (MATTR-50 = 0.785 vs. 0.765). Narrative-evidence cues occurred more frequently under MKO conditioning (40.0% vs. 10.0%, *p* < 0.001), while, among implicit questions, inference-oriented cues occurred in 36.1% of MKO-conditioned scaffolds compared with 2.8% of generic scaffolds (*p* =0.002). In contrast, question-specific reference did not differ significantly, and MKO conditioning did not reduce lexical answer overlap.

**Discussion:**

These findings demonstrate the feasibility of using structured task metadata to organize AI-generated scaffolding and show that metadata conditioning is associated with measurable differences in scaffold realization. The study contributes a transparent, task-sensitive framework for shifting AI-supported learning from answer provision toward structured guidance, while recognizing that learner-level pedagogical effectiveness requires direct human evaluation.

## 1 Introduction

The rapid advancement of large language models (LLMs) has intensified a longstanding ambition in Artificial Intelligence in Education: to deliver instructional support that is not only scalable, but also adaptive and pedagogically meaningful (Holmes and Tuomi, 2022). This ambition is grounded in evidence demonstrating the potential effectiveness of tutoring and intelligent tutoring systems (Ma et al., 2014; Nickow et al., 2024; Steenbergen-Hu and Cooper, 2014). A comprehensive meta-analysis of randomized preK-12 tutoring interventions reports an average effect size of 0.288 standard deviations, with stronger effects observed under conditions of sustained engagement, structured delivery, and trained human support (Nickow et al., 2020). These findings underscore a critical challenge for educational AI: while high-quality instructional support is demonstrably effective, it remains difficult to scale through human provision alone.

However, the value of tutoring lies not merely in delivering correct answers, but in structuring learners’ reasoning processes. Research on intelligent tutoring systems (ITS) has consistently shown that systems capable of modeling learner needs and delivering adaptive guidance outperform both traditional instruction and non-adaptive digital tools (Ma et al., 2014). This body of work establishes a key principle: *the effectiveness of educational technology depends on its capacity to provide structured, individualized scaffolding rather than automated solution delivery*.

Despite this, the current wave of generative AI has shifted attention toward answer generation, explanation generation, and content production. While these capabilities are technologically impressive, they are pedagogically insufficient. Recent scholarship has cautioned that AI systems risk becoming “powerful tools in search of a learning theory,” emphasizing the need to align system behavior with established principles of teaching and learning (Chowdhury et al., 2025; Sinha, 2025). The central issue is therefore not whether LLMs can produce educationally relevant language, but whether they can be organized to support *meaningful learning processes* rather than merely complete tasks.

The present study addresses this challenge by proposing *AI as the More Knowledgeable Other (MKO) 2.0*, an MKO-inspired framework that conceptualizes AI as a provider of task-sensitive scaffolding rather than direct answers. Drawing on sociocultural theories of learning and contemporary research on hint generation, MKO 2.0 uses structured task information to generate targeted guidance aligned with different comprehension demands. This approach responds to recent calls for stronger integration between educational theory, cognitive science, and NLP methods in the design of AI-supported learning systems (Jangra et al., 2025).

This need is particularly pronounced in narrative reading comprehension. Reading comprehension is inherently multi-dimensional, involving processes such as locating explicit information, generating inferences, tracking causal relationships, interpreting characters and emotions, and integrating information across a text. While current AI systems can answer comprehension questions or generate them, relatively few are designed to determine what kind of support is appropriate for a given comprehension demand. This gap is the focus of the present study.

Using metadata from the Quest Dataset, which was developed with foundational data from FairytaleQA (Xu et al., 2022), this study investigates whether question-level features, such as local versus summary scope, explicit versus implicit evidence status, and narrative attributes including action, causality, character, feeling, outcome, prediction, and setting, can be systematically transformed into task-sensitive scaffolds. The Quest Dataset was developed for automated question generation and was informed by foundational FairytaleQA data, but the present study repurposes its structured annotations as signals for scaffold generation. In doing so, the study advances a distinct research question: not whether AI can generate correct answers or plausible questions, but whether structured task information can be used to generate instructional guidance that varies with the reasoning demands represented in a question. This shift from answer production toward task-sensitive guidance constitutes the central contribution of the MKO 2.0 framework.

## 2 Literature review

### 2.1 From answer delivery to instructional guidance

A persistent limitation in contemporary AI-supported learning systems is their prioritization of *task completion over learning support*. This issue spans multiple subfields, including automatic question generation, question answering, LLM-based feedback, and conversational tutoring. Across these domains, technical progress has often outpaced pedagogical specificity (Kasneci et al., 2023; Kurdi et al., 2020).

The automatic question generation (AQG) literature illustrates this imbalance clearly. A systematic review of 93 studies found that while significant advances have been made in generating educational questions, most systems focus on assessment production rather than broader instructional functions such as feedback, scaffolding, or adaptive support (Kurdi et al., 2020). Consequently, the field has become increasingly effective at generating questions, but less capable of supporting learners as they engage with those questions.

This limitation persists in LLM-based approaches. While LLMs have demonstrated strong capabilities in generating explanations, feedback, and conversational responses, recent work emphasizes that educational applications impose requirements that extend beyond general notions of helpfulness (Xiao et al., 2023). In particular, systems must align their outputs with instructional intent, not merely linguistic fluency.

The problem becomes more pronounced in reading comprehension contexts. Different question types impose distinct cognitive demands: character questions require role identification, causal questions require reasoning about events, prediction questions require forward inference, and summary questions require integration across the text. Uniform responses may be insufficiently sensitive to these different comprehension demands. This creates a rationale for examining task-sensitive scaffolds that vary according to the reasoning represented in a question rather than providing the same form of guidance across tasks.

Recent work on inference-based question generation reinforces this concern. Although LLMs can produce fluent and plausible questions, they frequently fail to align outputs with intended reasoning types, highlighting a gap between linguistic quality and pedagogical precision (Jangra et al., 2025). This suggests that effective educational AI must explicitly model instructional logic, rather than relying on generative capability alone.

The same concern appears in recent work on LLM tutoring. Puech et al. (2025) argue that current LLMs are primarily trained to be helpful assistants, and therefore often reveal solutions too quickly or fail to sustain richer multi-turn pedagogical interactions. They introduce the problem of pedagogical steering, showing that if LLMs are to function as tutors, they must be deliberately guided toward effective instructional strategies rather than left to default answer-giving behavior. Their results support a crucial premise for the present study: in education, raw helpfulness is not always helpful. Sometimes, the pedagogically correct response is not to answer, but to scaffold (Puech et al., 2025).

### 2.2 Scaffolding, hints, and the More Knowledgeable Other

The theoretical foundation of this study lies in the literature on scaffolding and guided learning. Within this tradition, effective support is conceptualized not as generic assistance, but as contingent, adaptive, and minimally sufficient guidance that enables learners to perform tasks beyond their current independent capability while maintaining their active cognitive engagement, consistent with the notion of the Zone of Proximal Development (Belland, 2013; Van de Pol et al., 2010; Vygotsky, 1978; Wood et al., 1976).

Research on computer-based scaffolding emphasizes that high-quality support should structure learner reasoning, direct attention to relevant features, and sustain engagement without replacing cognitive effort (Sinha, 2025). This principle aligns closely with the concept of hints, which are designed to guide learners toward solutions while preserving their active participation in the task.

Recent scholarship further refines this perspective. A comprehensive survey of hint generation highlights the importance of integrating educational theory with NLP techniques, arguing that effective hints must balance support and independence while avoiding answer leakage (Jangra et al., 2025). This reinforces the need for systems that are not only capable of generating language, but also capable of selecting appropriate instructional strategies.

The concept of the More Knowledgeable Other provides a theoretical lens for organizing these ideas. Within sociocultural accounts of learning, a more knowledgeable participant can support performance beyond what a learner can accomplish independently (Vygotsky, 1978), while scaffolding research further emphasizes contingent support and the gradual transfer of responsibility to the learner (Van de Pol et al., 2010; Wood et al., 1976). Translating selected principles of this tradition into AI design suggests that systems should provide guidance that is sensitive to task demands, avoid unnecessary answer revelation, and leave responsibility for completing the task with the learner (Belland, 2013, 2017). MKO 2.0 therefore adopts an MKO-inspired approach in which structured task information is used to condition scaffold generation rather than treating AI as equivalent to a human More Knowledgeable Other. This orientation is consistent with recent work emphasizing the need to deliberately steer LLMs toward instructional strategies rather than relying on their default helpful-assistant behavior (Puech et al., 2025). The framework is therefore designed to leave responsibility for completing the comprehension task with the learner; learner agency itself is not evaluated in the present study.

### 2.3 Reading-comprehension AI and educational NLP

#### 2.3.1 Narrative comprehension datasets

The development of educationally grounded datasets has created new opportunities for integrating NLP and reading education. FairytaleQA (Xu et al., 2022) is particularly notable because it was designed to capture fine-grained narrative comprehension skills, including explicit and implicit reasoning across multiple narrative dimensions. Unlike many QA datasets, it incorporates expert-informed annotations that reflect pedagogically meaningful distinctions.

These datasets do more than provide benchmarks for question answering systems; they encode instructional structure. Narrative attributes such as causality, character, and prediction can be interpreted not only as classification labels, but also as task descriptors that may inform the type of reasoning support associated with a comprehension question. However, despite growing interest in educational NLP, the potential of these metadata structures for scaffold generation and pedagogically aligned instructional support remains comparatively underexplored (Jangra et al., 2025; Kurdi et al., 2020).

#### 2.3.2 Question generation and its limitations

While AQG research has advanced significantly, its focus remains largely on item generation rather than learner support. Even in recent LLM-based work, metadata is typically used to control question form rather than scaffold design. This results in a gap between assessment generation and instructional guidance, limiting the pedagogical utility of these systems (Kurdi et al., 2020). Recent work increasingly emphasizes that effective educational use of LLMs depends not only on their generative capabilities but also on mechanisms that structure or steer generation toward specified instructional strategies (Li et al., 2026; Puech et al., 2025; Ma et al., 2025). MKO 2.0 contributes to this direction by using explicit task characteristics to condition scaffold generation rather than relying solely on the default behavior of the language model.

#### 2.3.3 LLMs in education

Recent reviews highlight both the promise and limitations of LLMs in educational contexts. While these models enable scalable generation of explanations and feedback, challenges remain regarding reliability, pedagogical alignment, and integration into learning processes (Chowdhury et al., 2025; Mannekote et al., 2024; Shi et al., 2026). Recent research has also extended the use of LLMs beyond answer generation toward formative feedback. For example, Morris et al. (2025) examined LLM-generated formative feedback on student-authored summaries within intelligent textbooks, illustrating the potential of generative models to support learner-produced responses rather than simply provide answers. Extending this direction, Reddig et al. (2025) investigated the generation of in-context, personalized feedback for intelligent tutoring systems using large language models, highlighting the growing interest in integrating LLM-generated guidance into structured tutoring environments. However, the capacity to generate educational feedback does not necessarily mean that an LLM will adopt an appropriate instructional strategy. Work on pedagogical steering demonstrates that general-purpose LLMs do not inherently function as effective tutors and may provide solutions too readily when their behavior is not explicitly structured for instructional purposes (Puech et al., 2025). This reinforces the need for frameworks that deliberately structure generative behavior according to instructional or task-related information. MKO 2.0 addresses this need by using explicit task characteristics to condition scaffold generation rather than relying solely on the default behavior of the language model.

### 2.4 The present study

Despite these contributions, the literature reveals several gaps relevant to the present study. First, technical advances in educational AI do not by themselves establish theoretically grounded instructional support. Second, research on educational question generation has concentrated primarily on generating and evaluating questions, leaving scaffold generation comparatively underexplored (Jangra et al., 2025; Kurdi et al., 2020). Jangra et al. (2025), for example, identify effective hint generation as an important bridge between research in education, cognitive science, AI, and NLP. Third, linguistic fluency does not necessarily ensure that generated guidance reflects the particular demands represented in an educational task. Finally, recent work on pedagogical steering demonstrates that LLMs may require explicit instructional structure rather than relying on default answer-oriented behavior (Puech et al., 2025).

The present study addresses these issues by proposing a shift from descriptive metadata to instructional signals for scaffold generation. Rather than using question attributes solely for dataset organization or question generation, MKO 2.0 uses them to condition the selection and realization of scaffolds. The framework combines the transparency of rule-based generation with the flexibility of LLM-based generation and examines whether structured task information is associated with systematic differences in scaffold realization.

In this sense, MKO 2.0 is an MKO-inspired, task-sensitive framework in which structured question metadata is used to organize instructional guidance. The study therefore asks not whether AI can simply produce correct or fluent outputs, but whether task descriptors can be operationalized to generate different forms of guidance for different comprehension demands. Because the framework conditions support on task characteristics rather than individual learner states, it should be understood as task-sensitive rather than learner-adaptive.

Although the present study utilizes a narrative-comprehension dataset originally developed for younger readers, its broader contribution concerns the instructional architecture proposed for AI-supported learning rather than the age specificity of the source material. The principle of using structured task information to condition scaffold generation could be investigated in other educational contexts, including higher education, disciplinary reading, academic tutoring, and scaffolded reasoning. The applicability of MKO 2.0 beyond narrative comprehension, however, remains to be established empirically.

## 3 Method

### 3.1 Research design

This study employed a design-and-evaluation methodology to investigate whether structured task metadata can be operationalized as instructional signals for AI-generated scaffolding in narrative comprehension. The study conceptualized MKO 2.0 as an MKO-inspired computational framework rather than as a claim that an artificial intelligence system reproduces the interpersonal role of a human More Knowledgeable Other. The central design objective was to examine whether information about the reasoning demands of a comprehension question could systematically condition the form of support generated for that question.

The study comprised two related components. (1) a deterministic rule-based scaffold generator was developed to operationalize three dimensions of question metadata: the primary narrative-comprehension attribute (attribute1), explicit versus implicit evidence requirements (ex_or_im), and local versus summary comprehension scope (local_or_sum). Each dimension contributed separately to scaffold construction, allowing the rule-based implementation to serve as a transparent realization of the proposed task-to-scaffold mapping. (2) the framework was extended to an LLM-based experiment in which a narrative-grounded generic LLM condition was compared with an MKO-conditioned LLM condition. Both conditions received the same narrative context and comprehension question, while the MKO-conditioned condition additionally received the structured task metadata. This paired design allowed the analysis to examine whether metadata conditioning altered scaffold realization beyond what was produced by a generic narrative-grounded LLM.

The study therefore evaluates task-sensitive scaffold generation rather than learner-level adaptation. No learner profiles, interaction histories, performance states, or learning outcomes were modeled. Similarly, because the study did not involve learners or independent human raters, the evaluation does not establish pedagogical effectiveness. Instead, it examines the computational feasibility of metadata-conditioned scaffolding and observable characteristics of the generated outputs, including linguistic realization, narrative-evidence cues, task-sensitive inference language, and lexical overlap with reference answers.

### 3.2 Dataset and narrative context

The study used *The Quest Dataset*,^1^ a publicly available dataset developed for the Learning Agency Lab's *Quest for Quality Questions* initiative. The analytical file contained 6,989 question-answer pairs annotated with structured information about narrative-comprehension tasks. The dataset is related to the educational orientation of FairytaleQA, a narrative-comprehension resource developed from 278 children's stories and containing 10,580 expert-authored explicit and implicit questions across seven narrative-element or relation categories (Xu et al., 2022). FairytaleQA was explicitly designed around fine-grained narrative-comprehension distinctions and supports both question-answering and educational question-generation research.

The Quest data used in the present study contained the fields pair_id, source_title, cor_section, question, answer, local_or_sum, attribute1, attribute2, and ex_or_im. Three metadata dimensions were used directly in scaffold generation. The first, attribute1, identified the primary narrative-comprehension category: action, causal relationship, character, feeling, outcome resolution, prediction, or setting. The second, ex_or_im, distinguished questions whose answers were explicitly represented in the narrative from those requiring implicit inference. The third, local_or_sum, distinguished questions focused on a relatively localized portion of the narrative from summary questions requiring broader integration.

These dimensions were treated as task descriptors rather than direct measurements of individual learner need. For example, a causal-relation label indicates that a question requires reasoning about relationships between events; it does not establish that a particular learner personally requires causal scaffolding. This distinction was maintained throughout the study to avoid conflating task-sensitive support with learner-adaptive instruction. Narrative context was obtained from the accompanying source_texts.csv file, which contained source_title, cor_section, and narrative text. Question records were linked to their corresponding narrative sections using source_title and cor_section. Because some questions referenced multiple sections, section identifiers were parsed separately and the corresponding text segments were retrieved in their listed order. Duplicate source-section keys were consolidated by concatenating the associated text fragments before construction of the retrieval lookup. This procedure resulted in successful narrative-context retrieval for all 6,989 question-answer instances (100%).

The use of narrative text represented an important component of the LLM experiment. Rather than asking the language model to generate scaffolds from the question and metadata alone, both LLM conditions were supplied with the narrative evidence associated with each question. This made it possible to distinguish narrative-grounded generic hint generation from narrative-grounded, metadata-conditioned scaffold generation. The present study repurposes the dataset from its question-generation orientation toward scaffold generation. Metadata labels are therefore operationalized as structured signals describing the demands of the task. This use does not assume that the metadata constitute learner models; rather, it investigates whether those annotations can provide a transparent basis for conditioning instructional support.

### 3.3 Data preparation

All computational procedures were implemented in Python within Google Colab. The Quest files were retrieved programmatically and loaded into pandas DataFrames. Column names were standardized, categorical fields were inspected for consistency, and the question-level records were linked to their corresponding narrative sections. Preliminary validation included inspection of missing values, text-length distributions, metadata frequencies, and the correspondence between question records and source-text identifiers. The final analytical dataset contained 6,989 question–answer pairs. Of these, 6,367 (91.1%) were classified as local and 622 (8.9%) as summary questions. With respect to evidence status, 5,262 (75.3%) were explicit and 1,727 (24.7%) were implicit.

The primary reasoning attribute contained seven categories: action (*n* = 2,204; 31.5%), causal relationship (*n* = 1,949; 27.9%), character (*n* = 792; 11.3%), outcome resolution (*n* = 682; 9.8%), feeling (*n* = 665; 9.5%), setting (*n* = 422; 6.0%), and prediction (*n* = 275; 3.9%). These seven categories constitute the primary scaffold families in the rule-based implementation. Scope and explicit/implicit status were treated as conditioning dimensions rather than additional scaffold categories.

For LLM evaluation, an attribute-stratified sample of 140 instances was drawn from the complete dataset. Twenty questions were randomly selected from each of the seven attribute1 categories using a fixed sampling seed (random_state = 42). This sampling strategy replaced the smaller exploratory sample used during initial development and ensured representation of every primary reasoning category. The sample was balanced by primary attribute, but not artificially balanced by explicit/implicit status or comprehension scope; these dimensions retained naturally occurring variation within the sampled attribute groups. The resulting sample contained 104 explicit and 36 implicit questions, and 121 local and 19 summary questions.

### 3.4 MKO 2.0: task-sensitive scaffolding framework

MKO 2.0 was operationalized as an MKO-inspired task-sensitive scaffolding framework in which structured characteristics of a comprehension task are translated into instructional guidance. The framework is based on the principle that different narrative-comprehension questions impose different reasoning demands and that scaffold generation can therefore be conditioned on those demands. The framework comprises four conceptually distinct stages (Figure 1). (1) task representation identifies the narrative context, question, primary reasoning attribute, evidence status, and comprehension scope. (2) instructional diagnosis interprets these metadata as task-level reasoning requirements. A causal question, for example, calls for attention to relationships among events, whereas a prediction question calls for reasoning about likely subsequent events. Explicit/implicit status indicates whether guidance should emphasize directly stated information or inferential combination of evidence, while local/summary scope indicates the breadth of narrative information associated with the task.

**Figure 1:**
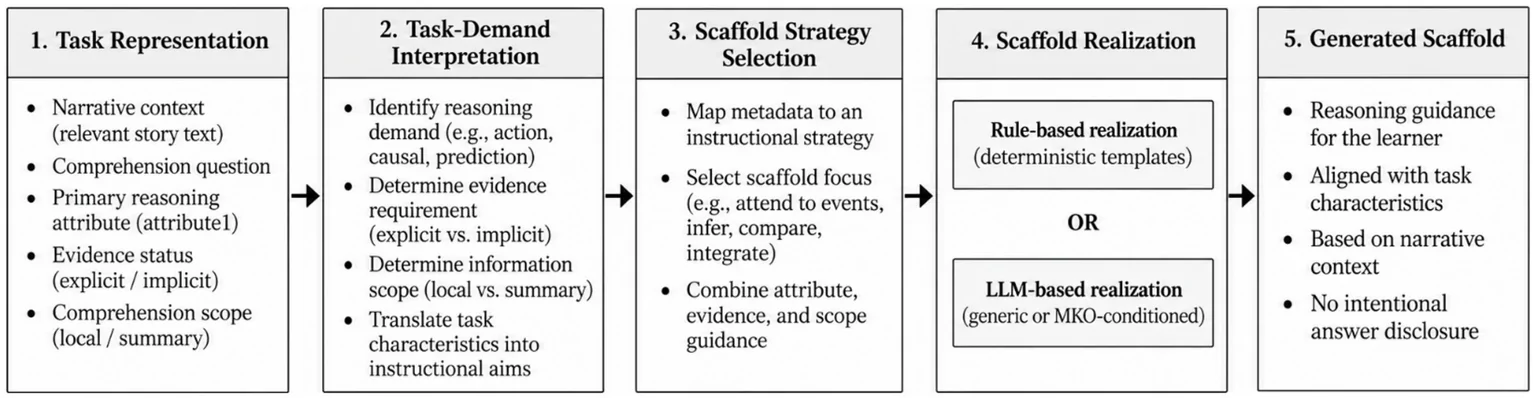
MKO 2.0 framework for task-sensitive scaffolding.

(3) Scaffold strategy selection maps these task characteristics to an instructional strategy. Importantly, the metadata describe the task rather than diagnosing an individual learner's cognitive state. (4) scaffold realization converts the selected strategy into a learner-facing hint. In the present study, realization was implemented through either deterministic rule composition or LLM generation.

The framework is therefore metadata-conditioned rather than learner-adaptive. Its purpose is not to infer what an individual learner knows, but to test whether structured task information can systematically influence the reasoning guidance expressed in an AI-generated scaffold.

### 3.5 Rule-based scaffold generation

The rule-based implementation provided a transparent operationalization of the MKO 2.0 framework. Unlike a single-label template system, the generator composed scaffolds from three task dimensions: primary reasoning attribute, explicit/implicit evidence status, and local/summary scope. The primary attribute determined the core reasoning strategy. Action questions directed attention to the key action associated with an event; causal-relationship questions prompted tracing events that could explain an outcome; character questions directed attention to actions, words, and relationships; feeling questions prompted interpretation of reactions and emotional evidence; outcome-resolution questions focused on how a problem or situation changed; prediction questions prompted consideration of likely subsequent events; and setting questions directed attention to where or when an event occurred.

A second component conditioned the scaffold on evidence status. For explicit questions, the scaffold directed attention toward information stated in the narrative. For implicit questions, it indicated that relevant clues might need to be combined to support an inference. A third component conditioned the scaffold on comprehension scope. For local questions, guidance focused attention on the narrative segment surrounding the event in question. For summary questions, guidance broadened attention across the relevant narrative.

The three components were concatenated to produce the final rule-based scaffold. Consequently, attribute1 determined the primary scaffold family, while ex_or_im and local_or_sum modified its realization. Across the full dataset, this procedure produced seven conceptual scaffold families and 28 distinct rule-based textual realizations. The rule generator was applied to all 6,989 instances. This implementation is deterministic and interpretable: identical combinations of the three metadata dimensions produce identical scaffold formulations. Its purpose is therefore not linguistic creativity but transparent implementation of task-to-scaffold mappings.

### 3.6 Experimental conditions

The comparative evaluation included three scaffold-generation conditions applied to the same attribute-stratified sample of 140 questions. The rule-based MKO condition used the deterministic procedure described in Section 3.5. It received the three structured metadata dimensions and generated a compositional scaffold according to predefined mappings. The generic LLM condition received the narrative context and question but did not receive the structured task metadata. It was instructed to provide one short hint that supported reasoning toward the answer, to use only information supported by the narrative, and not to state the final answer. The MKO-conditioned LLM condition received the same narrative context and question together with the question's reasoning attribute (attribute1), evidence status (ex_or_im), and comprehension scope (local_or_sum). It was instructed to use these task characteristics in shaping the scaffold while avoiding direct disclosure of the final answer.

The two LLM conditions were therefore evaluated on identical questions and narrative contexts. This paired design examined whether the MKO-conditioned generation condition was associated with observable differences in scaffold realization relative to narrative-grounded generic hint generation. The direct-answer and static generic-hint references used during conceptual development were not treated as experimental scaffold-generation conditions because they did not constitute comparable generation conditions. This distinction separates evaluation references from the architecture and empirical comparison of MKO 2.0.

### 3.7 LLM implementation and reproducibility

LLM generation was conducted through the OpenAI API in Google Colab using gpt-4.1-mini. The experiment used OpenAI Python package 2.54.0 under Python 3.13.15 on a Linux environment. Generation was conducted on September 19, 2026 (UTC). The temperature parameter was set to 0.0, and the maximum completion length was set to 120 tokens. One generation was obtained for each item under each LLM condition, producing 140 generic LLM hints and 140 MKO-conditioned LLM scaffolds, with no missing generations. Temperature 0 was used to reduce generation variability but was not assumed to guarantee deterministic output.

Both LLM conditions were grounded in the retrieved narrative context and were prohibited from intentionally providing the final answer. The generic condition used the following instructional structure:

*You are an educational tutor helping a student answer a reading comprehension question. Provide one short hint that helps the student reason toward the answer. Do not give the answer directly. Do not state the final answer. Use only information supported by the narrative*.

The user input supplied the narrative context and question.

The MKO-conditioned condition instructed the model to act as an MKO-inspired educational tutor supporting narrative comprehension, to scaffold rather than solve the task, to use only information supported by the narrative, and to use the supplied task metadata to shape the hint. Its input additionally specified the reasoning attribute, evidence status, and comprehension scope. No gold answer was included in either LLM generation prompt. Generated responses underwent no substantive post-processing beyond removal of leading and trailing whitespace. The prompts, model settings, generation code, and experimental outputs were preserved as part of the reproducibility record.

### 3.8 Evaluation strategy

The evaluation examined the computational characteristics and task-sensitive realization of the generated scaffolds rather than learner achievement or pedagogical effectiveness. Analyses were conducted on the attribute-stratified sample of 140 questions, with the rule-based, generic LLM, and MKO-conditioned LLM outputs compared descriptively. Because the two LLM conditions were generated for the same questions and narrative contexts, paired analyses were additionally used to examine differences between them.

#### 3.8.1 Scaffold length and lexical variation

Scaffold length was measured as the number of word tokens per hint. Unique word counts and per-hint type-token ratio (TTR) were calculated descriptively. Because both measures are sensitive to text length, moving-average type-token ratio with a 50-token window (MATTR-50) was used as the normalized corpus-level measure of lexical variation. For the two LLM conditions, paired differences in scaffold length were calculated for the same 140 questions. Because the paired differences were not assumed to satisfy parametric distributional requirements, a Wilcoxon signed-rank test was used to compare scaffold length between the generic and MKO-conditioned conditions. These measures characterized differences in linguistic realization and were not interpreted as measures of instructional quality.

#### 3.8.2 Lexical answer-overlap screening

Potential answer disclosure was examined using a lexical answer-overlap diagnostic. For each scaffold, tokenized scaffold text was compared with the corresponding reference answer, and the proportion of answer words appearing in the scaffold was calculated. The analysis also recorded whether any overlap occurred. This measure was treated as a screening diagnostic rather than an answer-leakage rate. Lexical overlap may reflect legitimate shared narrative terms, while answer-revealing paraphrases may occur without exact word overlap. Accordingly, the analysis was used to identify potentially answer-revealing lexical correspondence rather than to determine whether leakage had definitively occurred. For the paired LLM conditions, overlap was additionally classified as higher, lower, or unchanged under MKO-conditioned generation.

#### 3.8.3 Task-conditioning diagnostics

Exploratory diagnostics examined whether observable linguistic features differed between the generic and MKO-conditioned LLM outputs. Predefined lexical patterns were used to identify narrative-evidence cues, such as directions to consult the story, text, evidence, or relevant clues. Question-specific realization was examined by measuring whether a scaffold contained meaningful content words from its corresponding question after common function words were excluded. Because these features were binary and measured for paired outputs, differences between the two LLM conditions were examined using exact McNemar tests.

A more targeted analysis examined the explicit/implicit metadata dimension. Predefined inference-related expressions were used to identify inference-oriented language, and paired comparisons were conducted separately for questions annotated as explicit and implicit. A parallel exploratory diagnostic examined whether summary questions elicited explicit global-scope language.

These lexical diagnostics were interpreted as implementation indicators rather than measures of pedagogical quality. Their presence provides evidence of an observable linguistic realization, whereas their absence does not demonstrate that the corresponding instructional function is absent.

#### 3.8.4 Qualitative inspection

Selected scaffold pairs were inspected to contextualize the computational results and illustrate differences that lexical indicators alone could not fully capture. Examples were drawn from predefined comparison categories representing features detected in both conditions, one condition only, or neither condition, rather than selecting only cases favorable to MKO-conditioned generation. This inspection was descriptive and was not treated as independent expert validation. No learner, teacher, or expert ratings were collected.

### 3.9 Analytical procedure

The analytical workflow is summarized in Figure 2. Following acquisition and validation of the Quest data and associated narrative texts, rule-based scaffolds were generated for the full dataset (*N* = 6,989). An attribute-stratified sample of 140 questions was then constructed by randomly selecting 20 questions from each of the seven primary reasoning categories (random_state = 42). The same 140 questions and corresponding narrative contexts were used to generate generic and MKO-conditioned LLM scaffolds. The resulting outputs were examined using descriptive linguistic measures, lexical answer-overlap screening, paired comparisons, and task-conditioning diagnostics. This procedure allowed differences in scaffold realization to be examined while maintaining identical question-level inputs across the two LLM conditions.

**Figure 2:**
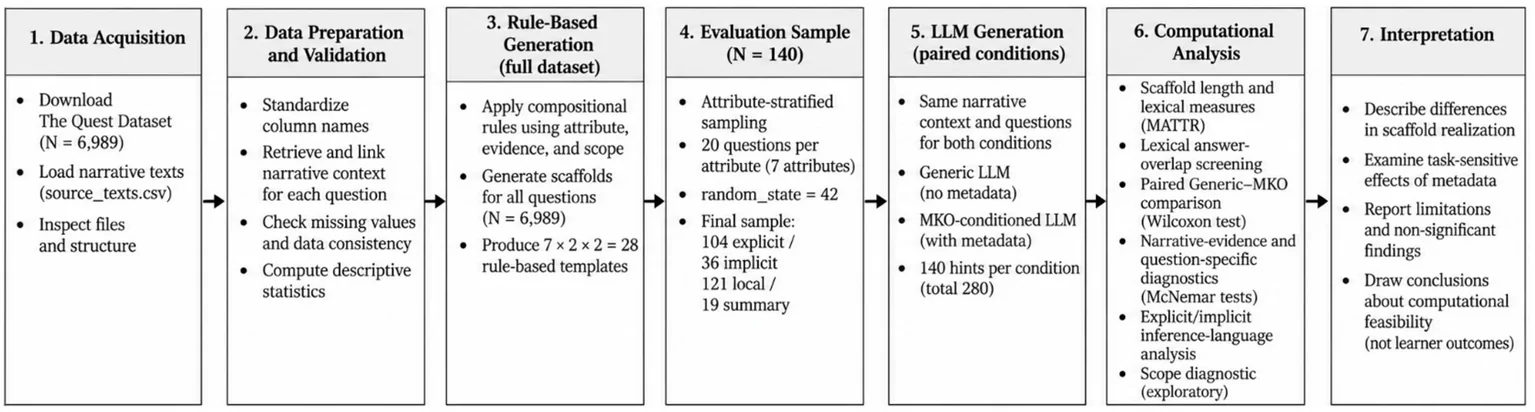
Analytical procedure for the MKO scaffolding study.

### 3.10 Trustworthiness and study limitations

Several design decisions were used to strengthen the transparency and trustworthiness of the computational evaluation. Rule-based scaffold generation was deterministic and explicitly linked to the three conditioning dimensions. Narrative context was successfully retrieved for all 6,989 question-answer pairs. The two LLM conditions used the same stratified sample, questions, and corresponding narrative contexts, while the rule-based condition was applied to the same 140 questions using the three structured metadata dimensions. The model configuration, prompts, sampling procedure, and generated outputs were retained for reproducibility. The evaluation also avoided treating metadata-derived lexical alignment as independent evidence of pedagogical quality.

The study nevertheless has important limitations. The framework is task-sensitive rather than learner-adaptive: it uses question metadata but does not model learner knowledge, performance history, or individual support needs. Furthermore, the LLM evaluation used an attribute-stratified sample of 140 questions rather than the complete dataset and was balanced by primary attribute rather than by scope or explicit/implicit status. Consequently, the latter analyses included fewer implicit (*n* = 36) and summary (*n* = 19) questions. The study evaluated a single LLM configuration with one generation per item. Setting temperature to 0 reduced generation variability but does not guarantee identical outputs across repeated API calls or model updates. In addition, the generic and MKO-conditioned prompts differed in both metadata availability and instructional framing; consequently, observed differences between these conditions cannot be attributed to metadata alone. Lexical indicators such as MATTR, answer overlap, and task-conditioning diagnostics characterize observable output properties but do not establish scaffold quality or learning effectiveness. In particular, lexical answer overlap cannot distinguish legitimate lexical correspondence from answer disclosure, and lexical-pattern diagnostics may not detect semantically equivalent expressions. The study did not include learners, teachers, or independent expert raters. The findings therefore provide evidence about computational feasibility, task-conditioned scaffold realization, and linguistic characteristics, not about improvements in comprehension, learner reasoning, or instructional effectiveness. Human evaluation and learner-based studies are required to establish those outcomes.

### 3.11 Reproducibility

To support reproducibility, the study retained the rule-generation procedure, data-preparation and narrative-retrieval code, attribute-stratified evaluation sample, exact LLM prompts, generated outputs, and analysis code. Together with the implementation settings reported in Section 3.7, these materials document the computational workflow from the underlying Quest data and associated narrative texts through scaffold generation and evaluation.

## 4 Results

### 4.1 Dataset characteristics and task distribution

The final analysis dataset comprised 6,989 question–answer pairs from the Quest Dataset. Narrative context was successfully retrieved for all 6,989 items, providing complete narrative coverage for subsequent LLM scaffold generation. Questions contained an average of 9.88 words (*SD* = 3.18), with a median of 10 and a range of 3–27 words. Answers averaged 7.02 words (*SD* = 5.92), with a median of 6 and a range of 1–69 words. These length measures are descriptive and should not be interpreted as indicators of task difficulty.

The dataset was predominantly local in scope (91.1%) and explicit in evidence status (75.29%). However, scope and evidence status were not distributed independently. Whereas 78.8% of local questions were explicit, only 39.4% of summary questions were explicit; 60.6% of summary questions were implicit. The principal dataset characteristics and scope-by-evidence distribution are summarized in Table 1.

**Table 1:** Dataset characteristics and distribution by comprehension scope.

Metric	Value
Number of question-answer pairs	6,989
Question length, M (SD)	9.88(3.18)
Question length, median (range)	10 (3–27)
Answer length, M (SD)	7.02 (5.92)
Answer length, median (range)	6 (1–69)
Local questions	6,367 (91.1%)
Summary questions	622 (8.9%)
Explicit questions	5,262 (75.29%)
Implicit questions	1,727 (24.71%)
Primary attribute categories	7

Scope × evidence status			
Scope	Explicit	Implicit	Total
Local	5,017	1,350	6,367
Summary	245	377	622
Total	5,262	1,727	6,989

The 6,989 questions were distributed across seven primary comprehension attributes (Table 2). Action was the most frequent category (*n* = 2,204, 31.54%), followed by causal relationship (*n* = 1,949, 27.89%). Evidence status also varied by attribute. Action, character, outcome-resolution, and setting questions were predominantly explicit, whereas feeling questions were more frequently implicit. Causal-relationship and prediction questions contained substantial representation of both evidence types. These distributions demonstrate that primary attribute and evidence status represent distinct dimensions of task structure.

**Table 2:** Distribution of primary comprehension attributes by evidence status.

Attribute	Total *n* (%)	Explicit	Implicit
Action	2,204 (31.54)	1,982	222
Causal relationship	1,949 (27.89)	1,100	849
Character	792 (11.33)	746	46
Outcome resolution	682 (9.76)	606	76
Feeling	665 (9.51)	283	382
Setting	422 (6.04)	417	5
Prediction	275 (3.93)	128	147
Total	6,989 (100.00)	5,262	1,727

### 4.2 Rule-based MKO scaffold generation

The rule-based MKO 2.0 implementation generated scaffolds for all 6,989 questions, with no missing outputs. Generation was deterministic and combined three task dimensions: primary comprehension attribute, explicit/implicit evidence status, and local/summary comprehension scope. Seven primary scaffold families corresponded to the seven comprehension attributes: action-focused reasoning (*n* = 2,204, 31.54%), causal reasoning (*n* = 1,949, 27.89%), character reasoning (*n* = 792, 11.33%), outcome-resolution reasoning (*n* = 682, 9.76%), emotion/feeling inference (*n* = 665, 9.51%), setting-focused reasoning (*n* = 422,422, 6.04%), and predictive reasoning (*n* = 275,275, 3.93%).

Evidence status and scope did not constitute additional scaffold families. Instead, they modified the realization of each attribute-based scaffold. Combining seven primary attributes with two evidence-status levels and two scope levels yielded 28 possible metadata-conditioned surface realizations (7 × 2 × 2). The rule-based implementation therefore provides evidence of complete and deterministic operationalization of the specified task-to-scaffold mapping across the full dataset. This result reflects implementation consistency rather than pedagogical effectiveness, because the scaffold mappings were defined by the generation rules themselves.

### 4.3 Comparative evaluation sample

Comparative evaluation used an attribute-stratified sample of 140 questions, with 20 questions randomly selected from each of the seven primary comprehension attributes. The same 140 questions were evaluated under all three conditions: rule-based MKO, generic narrative-grounded LLM, and MKO-conditioned LLM. The rule-based generator produced 140 metadata-conditioned scaffolds using each question's primary comprehension attribute, evidence status, and comprehension scope. The two LLM conditions received the same corresponding narrative contexts. Each condition produced 140 scaffolds with no missing outputs, yielding 420 outputs in total. Although sampling was balanced by primary attribute, evidence status and scope retained their naturally occurring distributions. The sample contained 104 explicit (74.29%) and 36 implicit (25.71%) questions, as well as 121 local (86.43%) and 19 summary (13.57%) questions.

### 4.4 Linguistic characteristics of the scaffold conditions

The three conditions differed in scaffold length and lexical realization (Table 3). Rule-based scaffolds were longest, averaging 37.57 words (*SD* = 33.27), followed by MKO-conditioned scaffolds at 23.61 words (*SD* = 4.17) and generic LLM scaffolds at 18.43 words (*SD* = 3.21). Raw unique-word counts followed the same ordering but were treated descriptively because they are affected by output length. Mean per-scaffold TTR was 0.800 for rule-based scaffolds, 0.908 for generic LLM scaffolds, and 0.883 for MKO-conditioned scaffolds. To reduce the influence of text length, corpus-level lexical variation was additionally assessed using MATTR with a 50-token moving window. MATTR-50 was 0.686 for the rule-based corpus, 0.765 for the generic LLM corpus, and 0.785 for the MKO-conditioned corpus.

**Table 3:** Linguistic characteristics of generated scaffolds.

Measure	Rule-based MKO	Generic LLM	MKO-conditioned LLM
*N*	140	140	140
Word count, *M* (*SD*)	37.57 (3.27)	18.43 (3.21)	23.61 (4.17)
Median word count	37	18	23
Mean unique words	30.11	16.71	20.79
Mean per-scaffold TTR	0.800	0.908	0.883
Corpus tokens	5,260	2,580	3,305
Corpus unique types	86	834	873
MATTR-50	0.686	0.765	0.785

As shown in Table 3, rule-based scaffolds were longer than both LLM conditions, whereas the two LLM corpora exhibited greater normalized lexical variation than the deterministic rule-based corpus. MATTR-50 was 0.686 for rule-based scaffolds, 0.765 for generic LLM scaffolds, and.785 for MKO-conditioned scaffolds. The difference between the two LLM conditions was comparatively small. These patterns describe differences in linguistic realization and should not be interpreted as differences in instructional quality.

The greater average length of MKO-conditioned relative to generic LLM scaffolds occurred across all seven attributes: action (24.55 vs. 18.30), causal relationship (24.35 vs. 19.15), character (22.45 vs. 18.45), feeling (23.25 vs. 19.70), outcome resolution (22.95 vs. 17.60), prediction (25.35 vs. 18.75), and setting (22.35 vs. 17.05). Rule-based scaffolds were also longer than both LLM conditions within every attribute.

### 4.5 Paired generic-MKO LLM comparison

Because the generic and MKO-conditioned LLM conditions generated scaffolds for identical questions and narrative contexts, question-level paired comparisons were possible. MKO-conditioned scaffolds contained an average of 5.18 additional words relative to their generic counterparts (median paired difference = 5.00, *SD* = 4.81). They were longer in 115 of 140 pairs (82.14%), equal in length in 13 (9.29%), and shorter in 12 (8.57%). A Wilcoxon signed-rank test indicated a significant paired difference in output length, *W* = 356.5, *p* < 0.001. MKO-conditioned scaffolds also contained an average of 4.09 additional unique words, whereas their per-scaffold TTR was slightly lower (mean paired difference = −0.025; median = −0.021) (Table 4). Corpus-level MATTR-50, reported above, was.785 for MKO-conditioned and.765 for generic outputs.

**Table 4:** Paired comparison of generic and MKO-conditioned LLM scaffolds.

Measure	Mean paired difference (MKO - Generic)	Median paired difference
Word count	+5.179	+5.000
Unique words	+4.086	+4.000
Per-scaffold TTR	−0.025	−0.021
Lexical answer overlap	−0.001	0.000

*N* = 140 paired questions. Positive values indicate higher values in the MKO-conditioned condition.

The observed length difference indicates greater elaboration under MKO conditioning; it does not establish that longer scaffolds are instructionally superior.

### 4.6 Lexical answer-overlap screening

Lexical answer overlap was examined as a screening measure of correspondence between scaffold language and the reference answer. Mean overlap was 0.006 (*SD* = 0.036) for rule-based scaffolds,.091 (*SD* = 0.191) for generic LLM scaffolds, and.090 (*SD* = 0.181) for MKO-conditioned scaffolds. Median overlap was zero in all three conditions (Table 5).

**Table 5:** Lexical answer-overlap screening across scaffold conditions.

Condition	*M*	SD	Median	Maximum	Any overlap, *n* (%)
Rule-based MKO	0.006	0.036	0.000	0.286	4 (2.86%)
Generic LLM	0.091	0.191	0.000	1.00	39 (27.86%)
MKO-conditioned LLM	0.090	0.181	0.000	1.00	42 (30.00%)

*N* = 140 per condition. Lexical answer overlap is a screening measure and should not be interpreted as a confirmed rate of semantic answer disclosure.

The paired LLM comparison showed essentially no mean difference in overlap (MKO-Generic = −0.001). MKO-conditioned overlap was higher in 21 pairs (15.00%), lower in 19 (13.57%), and identical in 100 (71.43%). Thus, MKO conditioning did not reduce lexical answer overlap relative to generic generation. Exact lexical correspondence can occur without revealing an answer, while semantically revealing language may occur without exact lexical overlap; the measure therefore should not be interpreted as a direct estimate of answer leakage.

### 4.7 Task-conditioning diagnostics

Because the task-conditioning diagnostics were designed to examine changes associated with adding MKO metadata conditioning to narrative-grounded LLM generation, these paired analyses focused on the generic and MKO-conditioned LLM conditions. The deterministic rule-based condition was evaluated separately through implementation coverage and the three-condition linguistic and answer-overlap comparisons reported above. Exploratory diagnostics examined whether observable linguistic features differed between generic and MKO-conditioned outputs. The analyses focused on narrative-evidence cues, question-specific reference, inference-oriented language associated with evidence status, and global-scope expressions associated with summary questions.

Narrative-evidence cues occurred in 40.0% of MKO-conditioned scaffolds compared with 10.0% of generic scaffolds. The paired transition pattern, 44 MKO-only cases compared with two generic-only cases, was significant by exact McNemar test (*p* < 0.001). Question-specific reference was common in both conditions, occurring in 89.3% of generic and 93.6% of MKO-conditioned outputs (Table 6). Mean question-word overlap was.384 and.492, respectively. The binary paired difference was not significant (*p* = 0.180), indicating that question specificity was not unique to MKO conditioning.

**Table 6:** Task-conditioning diagnostics for generic and MKO-conditioned LLM scaffolds.

Diagnostic	Generic LLM	MKO-conditioned LLM	Generic only	MKO only	Both	Neither	Exact McNemar *p*
General reasoning cue	107 (76.4%)	59 (42.1%)	54	6	53	27	<0.001
Narrative-evidence cue	14 (10.0%)	56 (40.0%)	2	44	12	82	<0.001
Question-specific reference	125 (89.3%)	131 (93.6%)	4	10	121	5	0.180
Inference cue: implicit (*n* = 36)	1 (2.8%)	13 (36.1%)	1	13	0	22	0.002
Inference cue: explicit (*n* = 104)	2 (1.9%)	3 (2.9%)	2	3	0	99	1.00
Global-scope cue: summary (*n* = 19)	0 (0%)	0 (0%)	0	0	0	19	–

Except where subgroup sample sizes are specified, *N* = 140 paired questions. “Generic only” and “MKO only” represent discordant pairs. Diagnostics were based on predefined lexical patterns and may not identify semantically equivalent expressions. The general reasoning-cue diagnostic is broad and does not measure the quality or appropriateness of reasoning support.

Differences in inference-oriented language were most apparent among implicit questions. Among the 36 implicit questions, inference-oriented language occurred in 13 MKO-conditioned scaffolds (36.1%) compared with one generic scaffold (2.8%), *p* = 0.002. Among the 104 explicit questions, inference-oriented language was uncommon in both conditions, three MKO-conditioned outputs (2.9%) and two generic outputs (1.9%), with no significant paired difference (*p* = 1.000). The broad general reasoning-cue diagnostic showed the opposite descriptive pattern, with cues detected in 76.4% of generic outputs and 42.1% of MKO-conditioned outputs (*p* < 0.001). Because this diagnostic identifies broad lexical expressions rather than the type, appropriateness, or quality of reasoning guidance, it is not interpreted as evidence that either condition provided superior reasoning support. The predefined global-scope diagnostic detected no scope-specific expressions in either LLM condition among the 19 summary questions. This null result provides no lexical evidence of distinct summary-scope realization. It may reflect limited scope-conditioned realization, limited sensitivity of the predefined diagnostic, or both; the analysis cannot distinguish between these explanations.

### 4.8 Systematic inspection of paired examples

The computational diagnostics were complemented by systematic qualitative inspection of paired generic and MKO-conditioned scaffolds (Table 7). Cases were organized according to predefined feature-transition categories, feature present in both outputs, only in the generic output, only in the MKO-conditioned output, or in neither, rather than selected solely because they favored one condition. The purpose was to examine how quantitatively detected features were realized in individual scaffolds and to identify cases in which predefined lexical diagnostics might not capture semantically similar guidance. No expert or learner ratings were assigned.

**Table 7:** Systematically selected examples of generic and MKO-conditioned scaffold realization.

Classification	Attribute	Evidence	Scope	Question	Generic LLM scaffold	MKO-conditioned scaffold
MKO-only cue detected	Causal relationship	Explicit	Local	Why couldn't the mother see the house?	Focus on what happened to the mother's eyes as she wept and how that might affect her ability to see.	Think about what happened to the mother's eyes and how it might affect her ability to see the house.
MKO-only cue detected	Feeling	Implicit	Local	How did Hase-Hime feel when she saw her father?	Focus on her initial reaction and the emotions she expressed through her words and actions.	Think about how Hase-Hime's reaction—being unable to speak or move, then bursting into tears—shows her emotions when she saw her father. What feelings might cause such a response?
Generic-only cue detected	Outcome resolution	Explicit	Local	What happened when the mistress fought on the meadow before the city?	Think about the outcome of the battle between the mistress with her followers and the dragon Tschauara's forces.	Focus on what the narrative says about the battle's result and how it affected the mistress's situation afterward.
Cue detected in both	Causal relationship	Explicit	Local	Why did the youth not want to eat the food?	Think about why the youth felt about being in the rat's place and what the food looked like to him.	Think about how the youth felt about being in the rat's place and what the food looked like to him—how might that affect his desire to eat?
Cue detected in neither	Setting	Explicit	Local	Where did Yuan Dschau return to?	Look for the place mentioned that Yuan Dschau went back to after finding his family situation disappointing.	Look for the place mentioned in the narrative where Yuan Dschau went back after finding his family situation.

Examples were drawn from predefined transition categories used in the systematic qualitative inspection. Classification refers to the diagnostic feature used for case organization and is not an expert rating of scaffold quality.

The paired examples illustrate that differences between conditions were not uniform. In some cases, MKO conditioning produced more explicit inferential or narrative-oriented guidance, whereas in others the generic scaffold contained comparable or more explicit reasoning language. Question-specificity was evident in both conditions, consistent with the high quantitative rates of question-specific reference. The examples therefore complement, rather than replace, the paired diagnostic analyses and do not constitute evidence of superior instructional quality.

Across the three scaffold-generation conditions, the results identified differences in linguistic realization and task-related lexical features. The deterministic rule-based implementation generated scaffolds for all 6,989 questions using seven primary scaffold families modified by evidence status and scope. Within the common 140-question evaluation sample, rule-based scaffolds were longer than both LLM conditions, whereas the two LLM corpora showed greater normalized lexical variation. MKO-conditioned LLM scaffolds were longer than their paired generic counterparts for 82.14% of questions (*W*  = 356.5, *p* < 0.001), although greater length should not be interpreted as greater instructional quality.

The task-conditioning diagnostics showed a mixed pattern. Narrative-evidence cues occurred more frequently in MKO-conditioned than generic scaffolds (40.0% vs. 10.0%, *p* < 0.001), and among implicit questions, inference-oriented language occurred more frequently under MKO conditioning (36.1% vs. 2.8%, *p* = 0.002). In contrast, question-specific reference was common in both conditions and did not differ significantly (*p* = 0.180). The global-scope diagnostic detected no scope-specific cues in either condition, and MKO conditioning did not reduce lexical answer overlap relative to generic generation. These results demonstrate the feasibility of operationalizing structured task metadata for scaffold generation and identify measurable differences between generic narrative-grounded and MKO-conditioned LLM scaffold realization. The clearest observed differences concerned scaffold elaboration, narrative-evidence language, and inference-oriented language among implicit questions. These findings concern generated scaffold characteristics and task-conditioned realization; they do not establish improved comprehension, learner agency, or pedagogical effectiveness, which require independent human and learner-based evaluation.

## 5 Discussion

The findings of this study support a shift in educational AI from answer generation toward structured instructional guidance. The MKO 2.0 framework demonstrates that question metadata can be operationalized as instructional signals for scaffold generation rather than used only as descriptive labels. Across the full dataset (*N* = 6,989), the rule-based system generated seven primary scaffold families and 28 surface realizations by combining comprehension attribute, explicit/implicit evidence status, and local/summary scope. This provides evidence for the feasibility of translating structured task information into differentiated scaffolding strategies. However, MKO 2.0 should be understood as an MKO-inspired, task-sensitive computational framework, rather than as an artificial replacement for a human More Knowledgeable Other. Traditional scaffolding depends on contingent support, fading, and transfer of responsibility in response to learner performance (Van de Pol et al., 2010; Wood et al., 1976). The present framework operationalizes task characteristics, not learner states, and is therefore more appropriately characterized as metadata-conditioned or task-sensitive than learner-adaptive.

A central contribution of the study is the reinterpretation of educational metadata as a resource for scaffold generation. Automatic question-generation research has primarily concentrated on producing and evaluating questions, while feedback generation has received comparatively less attention (Kurdi et al., 2020). Similarly, FairytaleQA demonstrates the value of fine-grained narrative-comprehension annotations for distinguishing explicit and implicit questions and different narrative elements and relations (Xu et al., 2022). MKO 2.0 extends this logic by using structured task descriptors to inform the type of support generated *after* a comprehension question has been posed. In this sense, metadata moves from a descriptive role to an instructional-control role. Importantly, the deterministic correspondence between metadata and rule-based scaffold families should not be interpreted as independent evidence of pedagogical effectiveness. Rather, it demonstrates transparent task-scaffold consistency within the computational design.

The comparison between the generic narrative-grounded and MKO-conditioned LLM conditions provides further evidence of task-sensitive scaffold realization. Across the same 140 questions and narrative contexts, MKO-conditioned scaffolds were longer than their generic counterparts in 82.14% of cases, with an average paired difference of 5.18 words (*W*  = 356.5, *p* < 0.001). Corpus-level MATTR was also somewhat higher for MKO-conditioned scaffolds (0.785) than for generic scaffolds (0.765), although the difference was modest and should not be interpreted as evidence of superior instructional quality. More substantively, narrative-evidence cues occurred in 40.0% of MKO-conditioned outputs compared with 10.0% of generic outputs (*p* < 0.001). Among implicit questions, inference-oriented cues occurred in 36.1% of MKO-conditioned scaffolds but only 2.8% of generic scaffolds (*p* = 0.002). These patterns are consistent with the intended function of metadata conditioning: evidence-status information was associated with observable differences in how guidance was linguistically realized.

At the same time, the results qualify any broad claim that metadata conditioning makes LLM scaffolds uniformly more context-sensitive or pedagogically superior. Question-specific reference was already highly prevalent in the generic narrative-grounded condition (89.29%) and increased only to 93.57% under MKO conditioning; the paired difference was not significant (*p* = 0.180). This suggests that supplying the narrative and question alone enables an LLM to produce substantial question-specific contextualization. The more distinctive contribution of MKO conditioning in the present results concerns particular forms of guidance, especially explicit reference to narrative evidence and inference-oriented language for implicit questions, rather than contextual specificity in general.

The answer-overlap analysis provides an additional qualification. Lexical overlap with reference answers was almost identical in the two LLM conditions (generic: *M* = 0.091; MKO-conditioned: *M* = 0.090), and MKO conditioning did not systematically reduce overlap. This result is important because the intended function of scaffolding is to support reasoning without simply providing the answer. However, lexical overlap is only a screening measure: legitimate scaffolds may share words with an answer, while semantically revealing hints may use different vocabulary. The finding therefore does not establish answer leakage, but it indicates that metadata conditioning alone cannot be assumed to reduce potentially answer-revealing language. More direct semantic or human evaluation is required.

The scope results were similarly mixed. Although local/summary scope was incorporated into the framework, the predefined global-scope diagnostic detected no relevant lexical cues in either LLM condition among the 19 summary questions. Consequently, the present findings do not provide observable lexical evidence that scope conditioning affected LLM scaffold realization. This null result may reflect limitations of the lexical diagnostic, but that explanation cannot be established from the present data. It therefore identifies scope-sensitive scaffolding as an area requiring stronger operationalization and evaluation.

The rule-based and LLM results illustrate complementary characteristics of the two approaches. The rule-based implementation offers transparency and deterministic control: each scaffold can be traced to an explicit combination of task descriptors. The LLM conditions provide greater generative variation and question-specific realization. Recent research on AI-assisted feedback similarly emphasizes the growing capacity of generative models to produce elaborated educational feedback while also highlighting the importance of grounding, transparency, and careful evaluation of whether generated feedback actually supports learning (Ba et al., 2025; Maier et al., 2026). A 2025 meta-analysis of prompting as digital scaffolding likewise found that the effectiveness of prompts varies according to how support is structured, reinforcing the importance of instructional design rather than treating prompts as interchangeable forms of assistance (Thomann and Deutscher, 2025). MKO 2.0 contributes to this direction by providing an explicit task representation that can structure generative support while retaining an interpretable connection between the comprehension task and scaffold strategy.

The findings also position the study within the broader development of LLM-based educational systems. Recent reviews report growing use of LLMs for tutoring and feedback but continue to identify concerns surrounding reliability, over-reliance, pedagogical integration, and the quality of generated support (Létourneau et al., 2025; Shi et al., 2026). The present study suggests that model capability alone is not the only relevant design consideration; the instructional information supplied to the model also matters. At the same time, the results caution against equating linguistic differences with learning benefits. Longer scaffolds, higher MATTR, or greater use of evidence-oriented language may characterize different forms of support, but none demonstrates that learners understand more, reason more effectively, or require less assistance.

Although the Quest data concern narrative comprehension, the broader contribution of MKO 2.0 lies in its instructional architecture rather than in the specific stories or learner age range represented by the underlying data. The general principle, representing task demands explicitly and using those representations to condition guidance, could potentially be investigated in disciplinary reading, academic tutoring, argumentation, and other reasoning-intensive learning activities. Such generalizability remains a proposition for future research rather than an empirical finding of the present study.

### 5.1 Contributions to theory and practice

This study contributes theoretically by providing a computational interpretation of MKO-inspired scaffolding in which structured task information informs scaffold selection without treating an LLM as equivalent to a human MKO. It also clarifies an important distinction between task-sensitive support and learner adaptation: the framework responds to properties of questions but does not diagnose an individual learner's knowledge or needs. This distinction is consistent with scaffolding theory, in which genuine contingent support depends on responsiveness to learner performance (Van de Pol et al., 2010).

Methodologically, the study demonstrates how existing educational metadata can be repurposed as generation controls. The three-condition design further separates deterministic rule-based generation, generic narrative-grounded LLM generation, and MKO-conditioned generation. This is important because the generic LLM results show that question-specific contextualization can arise from narrative grounding alone, whereas the evidence- and inference-related findings provide more specific indications of task-conditioned realization.

From a design perspective, the findings support combining interpretable instructional structure with generative flexibility. Rule-based generation provides transparent control over scaffold strategy, while LLM generation permits more varied realization. The results therefore suggest a design space in which structured pedagogical representations constrain or condition generative models rather than leaving instructional behavior entirely implicit in the model. This contribution concerns the design and computational plausibility of scaffolding; pedagogical effectiveness remains to be established through human evaluation.

### 5.2 Limitations and future directions

The present study has several limitations that should be considered when interpreting its findings. The study examined generated scaffolds rather than learner outcomes, and no learners, teachers, or independent expert evaluators assessed the outputs. The findings therefore cannot establish improvements in comprehension, engagement, learner agency, or pedagogical effectiveness. This distinction is particularly important because scaffolding is fundamentally contingent on learner performance and the transfer of responsibility from the more knowledgeable participant to the learner (Van de Pol et al., 2010; Wood et al., 1976). The scope of the LLM evaluation also warrants consideration. The comparison was conducted on an attribute-stratified sample of 140 questions, with 20 questions selected from each of the seven primary categories. Although this ensured representation across the full attribute taxonomy, evidence status and comprehension scope were not independently balanced: the sample included 36 implicit questions and 19 summary questions. In addition, the experiment used a single LLM configuration and one generation per item. A temperature setting of 0 increased repeatability but does not guarantee identical outputs across repeated API calls or future model updates. The findings should therefore be interpreted in relation to the specific model, configuration, and sample examined in this study.

The computational measures provide another boundary on interpretation. MATTR-50 captures normalized lexical variation rather than instructional quality, while lexical answer overlap serves only as a screening measure for potential answer disclosure. Similarly, the task-conditioning diagnostics rely on predefined lexical patterns and may fail to identify semantically equivalent expressions. This limitation was particularly apparent in the scope analysis, where the predefined diagnostic detected no global-scope cues in either LLM condition. Accordingly, these measures provide evidence about observable properties of scaffold realization rather than direct evidence of pedagogical quality.

Future research should extend the present computational evaluation through independent expert and learner studies. Such work could examine whether task-sensitive scaffolds improve comprehension, reasoning, transfer, help-seeking, or other learning outcomes relative to generic guidance. The framework could also be extended from task-sensitive generation to genuinely learner-contingent scaffolding, in which support changes in response to learner answers, misconceptions, or repeated difficulty. Recent work on pedagogical LLM tutors illustrates the potential of combining explicit scaffolding plans with information about learners’ evolving cognitive states to guide subsequent tutoring actions (Li et al., 2026). Such an extension would more closely approximate the contingency, fading, and transfer of responsibility central to established accounts of scaffolding (Van de Pol et al., 2010; Wood et al., 1976). Further studies should also compare multiple LLMs and repeated generations, incorporate semantic and human evaluation alongside lexical diagnostics, and examine whether the framework transfers beyond narrative comprehension to other educational tasks.

## 6 Conclusion

This study demonstrates the feasibility of using structured task metadata to organize AI-generated scaffolding for narrative comprehension, support task-sensitive scaffold generation and identify measurable differences in scaffold realization. They do not establish improvements in learner comprehension, engagement, or other learning outcomes, which require direct evaluation with learners. The rule-based implementation provided transparent and complete task-conditioned scaffold generation across 6,989 questions, while the paired LLM analysis showed that MKO-conditioned generation differed from generic narrative-grounded generation in scaffold elaboration, narrative-evidence language, and inference-oriented language for implicit questions. Other results were more limited: question-specific reference did not differ significantly, lexical answer overlap was essentially unchanged, and the scope diagnostic detected no difference.

Accordingly, the contribution of MKO 2.0 is not evidence that AI has become a human-like More Knowledgeable Other or that metadata-conditioned scaffolds are already pedagogically superior. Rather, MKO 2.0 provides an MKO-inspired computational architecture for translating structured task information into task-sensitive instructional guidance. The next step is to determine whether these computational differences translate into meaningful benefits for learners.

## Data Availability

The original contributions presented in the study are included in the article/supplementary material, further inquiries can be directed to the corresponding author.

## Footnotes

1
https://www.kaggle.com/datasets/julesking/the-quest-dataset/data
